# Green synthesis of nanoparticles using medicinal plants as an eco-friendly and therapeutic potential approach for neurodegenerative diseases: a comprehensive review

**DOI:** 10.3389/fnins.2024.1453499

**Published:** 2024-11-22

**Authors:** Rezvan Izadi, Seifollah Bahramikia, Vali Akbari

**Affiliations:** Faculty of Basic Sciences, Department of Biology, Lorestan University, Khorramabad, Iran

**Keywords:** amyloid, green synthesis, nanoparticles, neurodegenerative diseases, medicinal plants

## Abstract

Central nervous system disorders impact over 1.5 billion individuals globally, with neurodegenerative diseases such as Alzheimer’s, Parkinson’s, and Huntington’s diseases being particularly prominent. These conditions, often associated with aging, present debilitating symptoms including memory loss and movement difficulties. The growing incidence of neurological disorders, alongside a scarcity of effective anti-amyloidogenic therapies, highlights an urgent need for innovative treatment methodologies. Nanoparticles (NPs), derived from medicinal plants and characterized by their favorable pharmacological properties and minimal side effects, offer a promising solution. Their inherent attributes allow for successful traversal of the blood–brain barrier (BBB), enabling targeted delivery to the brain and the modulation of specific molecular pathways involved in neurodegeneration. NPs are crucial in managing oxidative stress, apoptosis, and neuroinflammation in ND. This study reviews the efficacy of green-synthesized nanoparticles in conjunction with various medicinal plants for treating neurodegenerative diseases, advocating for further research to refine these formulations for enhanced clinical applicability and improved patient outcomes.

## 1 Introduction

Neurodegenerative diseases (ND) is characterized by the gradual loss of neurons, structural and functional impairments in the brain and spinal cord, as well as cognitive and physical decline, ultimately leading to the direct and indirect demise of patients. The World Health Organization estimates that 50 million people worldwide are affected by neurodegenerative disorders – primarily characterized by motor neuron dysfunction and loss – and that number is expected to rise as our population ages (Scatena et al., 2007; Yavarpour-Bali et al., 2019; Bhattacharya et al., 2022). Numerous factors contribute to the development of ND, including the accumulation of amyloid proteins, intracellular or extracellular protein misfolding within the CNS, neuroinflammation, oxidative stress, neurotransmitter depletion such as butyrylcholine (BCh) and acetylcholine (ACh), and disruption of the blood–brain barrier (BBB) (Rasool et al., 2014; Maiti and Dunbar, 2018). Genetic predisposition plays a significant role in ND, alongside factors like excessive brain accumulation of metals such as copper (Cu), zinc (Zn), lead (Pb), and iron (Fe), mitochondrial dysfunction, and impaired redox reactions (Masoudi Asil et al., 2020; Sriramcharan et al., 2022). Additionally, certain chemicals like monoamine oxidase and cholinesterase contribute to the breakdown of dopaminergic and cholinergic synapses (Teibo John et al., 2020).

Conditions like Alzheimer’s diseases (AD), Parkinson’s diseases (PD), and Huntington’s diseases (HD) are closely linked to the aging process, manifesting symptoms such as memory loss, movement impairments, and speech and breathing difficulties. (Gitler et al., 2017; Bhattacharya et al., 2022). AD represents the most prevalent form of dementia, with the number of AD patients reaching 50 million by 2017. The disease has multifaceted causes, with abnormal amyloid *β* (Aβ) accumulation being a key factor (Kalimuthu et al., 2020; Huang et al., 2021). AD prevalence rises notably after age 65, with a marked exponential increase with advancing age. AD risk factors encompass familial history, head injuries, genetic factors (apolipoprotein E), gender (female), vascular conditions, and environmental influences. In familial AD cases, mutations in presenilin 1 and 2 genes are observed, accounting for 2-3% of AD instances and affecting individuals under 65 (Singh et al., 2010; Castellani et al., 2010). Loss of synapses in AD is associated with the accumulation of low-solubility Aβ species. Amygdala regions in AD patients have many plaques and neurofibrillary. Also, two types of Aβ plaques are seen in the brain parenchyma along with tau inclusions. Most of these patients also have amyloid angiopathy (Castellani et al., 2010; Dugger and Dickson, 2017).

Oxidative damage triggered by mitochondrial dysfunction (induced by Aβ42) and glial activation leads to cytotoxicity and calcium overload (Emerit et al., 2004). The integrity of brain cell support and transport systems relies on the tau protein’s proper structure and function. In AD, the abnormal twisting of tau strands results in dysfunctional tangles within brain cells, disrupting the transport system and culminating in cell death (Agarwal et al., 2013). PD represents the most prevalent motor ND, affecting at least 1% of individuals over 70 years of age. Approximately 80% of PD patients develop dementia within two decades. Parkinson’s disease dementia (PDD) is characterized by deficits in short-term memory and decision-making functions, stemming from the degeneration of subcortical nuclei like the medial substantia nigra and the cholinergic nucleus basalis of Meynert. Dopamine deficiency in the striatum due to the loss of dopamine-producing cells is a hallmark of PD (Savitt et al., 2006; Edison et al., 2013; Irwin et al., 2013).

Oxidative stress and neuroinflammation are primary contributors to the death of dopaminergic neurons (Khazdair et al., 2020). The etiology of PD includes the accumulation of intracellular *α*-synuclein aggregates, reduced activity of mitochondrial complex 1, and telomere shortening (Hou et al., 2019). Additionally, PD is characterized by factors such as iron accumulation in the zona, elevated nitrogen levels in Lewy bodies, activation of the caspase cascade, and increased apoptosis (Emerit et al., 2004). Aβ is present in both PD and PDD, with significant Aβ levels observed in 40% of PDD cases (Edison et al., 2013).

HD is an autosomal dominant disorder affecting 4-10 individuals per 100,000 people. While typically manifesting between ages 30 and 50, HD can also onset as early as two or as late as over 80 in rare cases (Sandhir et al., 2014). HD leads to degeneration of the striatum, hypothalamus, and cerebral cortex, resulting in motor, cognitive, and behavioral impairments, weight loss, disruptions in circadian sleep rhythms, and autonomic nervous system dysfunction (Popovic and Brundin, 2006; Roos, 2010). HD is caused by misfolding of the huntingtin protein into its β form and post-translational modifications like phosphorylation. This abnormal protein disrupts cellular metabolism and mitochondrial function, generating atypical metabolites and markers of oxidative stress. Neuronal death in HD is associated with movement disorders, with disease progression influenced by environmental and genetic factors (Ross et al., 2014).

This study addresses the critical relevance of central nervous system disorders affecting over 1.5 billion individuals worldwide, particularly highlighting neurodegenerative diseases such as Alzheimer’s and Parkinson’s. While current literature acknowledges the urgent need for effective treatments, significant gaps remain in the application of nanoparticle (NP) technology derived from medicinal plants. Specifically, there is a lack of comprehensive research on the optimization of NP formulations for targeted delivery across the blood–brain barrier and their specific mechanisms in mitigating neurodegeneration. This study aims to address these gaps by systematically reviewing existing data and proposing refined NP strategies to enhance therapeutic efficacy. The main contributions of this research include advancing the understanding of green-synthesized nanoparticles in neuroprotection and offering a pathway for developing innovative treatment options with minimal side effects for affected patients.

## 2 Amyloids

Amyloids are fibrous, insoluble proteins resistant to protease degradation, forming aggregates in the cytoplasm of neurons, glia, parenchyma, and blood vessel walls as plaques or amyloid angiopathy. Amyloid plaques can manifest in various forms, including diffuse, dense-cored, classical, and cotton wool, which vary based on the type of amyloid, disease stage, and deposition site (Dugger and Dickson, 2017; Girigoswami et al., 2019). These amyloid deposits can lead to significant tissue damage and cell death (Ghosh et al., 2021). By binding to cell membranes, amyloid accumulations can disrupt membrane integrity, leading to increased cellular damage, oxidative stress, cytoskeletal alterations, organ dysfunction, and apoptosis (Ghosh and De, 2020). This membrane disruption is a key driver of amyloid-induced cytotoxicity (Wang et al., 2017).

Human amyloidosis involves over 20 amyloidogenic peptides and pathogenic proteins like Aβ, *α*-synuclein, Tau, and serum amyloid protein. Amyloidosis is categorized into systemic and local forms, with systemic involvement across multiple organs and local involvement in specific tissues (Wang et al., 2017; Ghosh and De, 2020). Amyloid deposition is common in individuals with a genetic disorder associated with apolipoprotein E (Dugger and Dickson, 2017).

## 3 Oxidative stress

Oxidative stress inflicts damage on cell proteins and lipid membranes, primarily driven by free radicals such as reactive oxygen species (ROS) and reactive nitrogen species (RNS) (Ríos et al., 2016; Chandran and Abrahamse, 2020). The nervous system is particularly susceptible to ROS due to its high oxygen consumption, limited antioxidant capacity, abundance of steroid lipids, and metal catalyst content (Sharifi-Rad et al., 2022). ROS plays a pivotal role in neurodegeneration in AD, PD, and HD, leading to mitochondrial dysfunction, neuroinflammation, and elevated levels of nuclear factor κB (NFκB) and insulin-like growth factor (IGF) (Ríos et al., 2016; Chandran and Abrahamse, 2020; Sharifi-Rad et al., 2022).

Edible oyster mushrooms are notable for their rich composition of bioactive compounds, such as phenolics, flavonoids, ascorbic acid, glycosides, tocopherols, polysaccharides, ergothioneine, and carotenoids. These compounds possess robust antioxidant properties that effectively combat free radicals, thereby playing a significant role in mitigating oxidative stress (Gupta et al., 2017). The administration of medicinal mushroom extracts has demonstrated potential in treating patients and offering protection against a variety of diseases, including neurodegenerative disorders (Chaturvedi et al., 2020).

Mitochondrial superoxide radicals impair movement within the brain, resulting in DNA damage and the onset of neurodegenerative diseases. Hydrogen peroxide (H_2_O_2_) is linked to excessive oxidation in nerve cells, with peroxisomes typically responsible for controlling superoxide radicals and H_2_O_2_ enzymatically. In AD, hyperphosphorylated tau proteins lead to catalase (CAT) and peroxidase depletion from peroxisomes, exacerbating oxidative stress (Chandran and Abrahamse, 2020; Sharifi-Rad et al., 2022).

## 4 Neuroinflammation

Neuroinflammation is a common feature of ND, characterized by elevated levels of cytokines and inflammatory markers in AD, PD, and HD. In neuroinflammation, the brain’s innate immune response triggers an increase in chemokine concentrations like interleukin-6 (IL-6), IL-1β, CC-motif ligand-2 and 5 (CCL-2 and 5), and CXC-motif ligand-1 (CXCL-1), promoting ROS and RNS production and enhancing BBB permeability (Fakhri et al., 2022).

Microglial cells, part of the brain’s mononuclear phagocyte system, play a crucial role in neuroinflammation. Inflammatory responses by microglia contribute to the demise of dopamine-producing cells. Microglia induce cell damage by releasing IL-1, 6, and 12, tumor necrosis factor (TNF-*α*), and nitric oxide (NO), stimulating amyloid precursor protein production, and elevating Aβ levels. Conversely, Aβ enhances microglial activation (Klegeris and McGeer, 2005; Edison et al., 2013). Cytokines released by microglia bind to neuronal receptors, activating apoptotic pathways (Sharifi-Rad et al., 2022).

## 5 Metal accumulation in the brain

Excessive accumulation of metals in the brain leads to oxidative damage, mitochondrial dysfunction, protein misfolding, impaired autophagy, neuroinflammation, and neuronal death (Yan et al., 2022).

In AD, calcium release from the endoplasmic reticulum disrupts memory, while the Cu, Zn, and Fe buildup enhances Aβ accumulation and triggers oxidative stress. Manganese accumulation inhibits glycolysis, causing toxicity and cytoskeletal disruption in HD, while abnormal copper-protein interactions contribute to HD development by affecting the huntingtin structure. Elevated Fe levels in PD lead to ferroptosis and the loss of dopaminergic neurons (Yan et al., 2022).

## 6 Acetylcholinesterase (AChE) and butyrylcholinesterase (BChE) enzymes

ACh is the essential natural brain substance that affects memory, speech, concentration, and logical reasoning. BCh is also effective on memory. BChE and AChE are in the group of serine hydrolases. BChE is mainly found in the white matter and areas effective in cognition and behavior. The main action of BChE and AChE is the simultaneous regulation of ACh. An increase in the level of AChE and BChE leads to the breakdown of ACh and BCH and reduces their stimulatory effects. In AD, the number of neurons expressing BChE is increased and is associated with the formation of amyloid plaques. Inhibition of AChE and BChE can reduce the accumulation of Aβ and the formation of nerve fibrils and increase the level of ACh (Darvesh, 2016; Gul et al., 2021).

## 7 Available treatments for AD, PD, and HD

Memantine enhances cholinergic signaling and inhibits glutamate overactivation through N-methyl-D-aspartate receptor inhibition in AD. Cholinesterase inhibitors like tacrine, donepezil, rivastigmine, and galantamine manage AD symptoms (Casey et al., 2010; Tang et al., 2023). Levodopa is a common PD treatment that stimulates dopamine receptors (Schapira, 2005).

**Figure 1:**
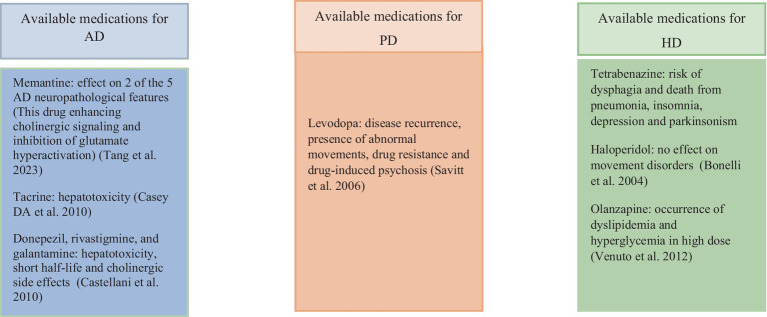
Complications and limitations of drugs used for the treatment of AD, PD and HD.

The drugs olanzapine and haloperidol help to manage Huntington’s disease by decreasing chorea symptoms, while tetrabenazine works by reducing dopamine levels in the brain. These medications are crucial in alleviating motor symptoms and improving the quality of life for individuals with HD (Bonelli et al., 2004). The rising prevalence of neurological disorders, coupled with the limited availability of anti-amyloidogenic drugs, underscores the urgent need for innovative treatment approaches (Ghosh and De, 2020). In addition, given the side effects associated with existing treatments (Figure 1), the utilization of medicinal plants for treating AD and other diseases has been under consideration (Ovais et al., 2018).

## 8 Medicinal plants

Medicinal plants harbor bioactive compounds with potent pharmacological properties and minimal side effects (Table 1) (Hassan et al., 2022; Mishrikoti et al., 2022). Different plant parts, such as flowers, seeds, fruits, roots, leaves, and bark, are used in disease treatment, with extraction methods varying based on compound characteristics. For this purpose, polar and nonpolar solvents and methods such as sonication, Soxhlet extraction, and heating under reflux are utilized (Chandran and Abrahamse, 2020; Mishrikoti et al., 2022). Secondary metabolites like sterols, polyphenols, lignans, flavonoids, alkaloids, triterpenes, and tannins from medicinal plants show efficacy in combating CNS-related diseases by targeting AChE, BChE, oxidative stress, and neuroinflammation (Gul et al., 2021; Bakrim et al., 2022).

**Table 1:** Some plants and herbal compounds effective on ND.

Medicinal plants	Mechanism of effect
*Verbascum phoeniceum*	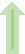 Inflammation and production of cyclooxygenase-1 (COX-1) and COX-2 (de Rus Jacquet et al., 2017)
*Boswellia serrata*	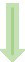 ACh 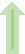 AChE levels Improving motor ability, improving memory (Nemat A.Z. Yassin et al., 2013)
White Tea	Inhibition of Aβ fibrillation (Li et al., 2019)
*Bacopa monnieri*	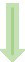 Longevity and GPx, SOD, CAT, and GSH 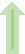 Irritability and insomnia, lipid peroxidation and protein oxidation Improving memory (Jyoti and Sharma, 2006; Goswami et al., 2011)
Curcumin	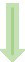 Body rotation, limb strength, muscle coordination, SOD, CAT, GSH and dopamine D2 binding Inhibition of AChE and inhibition of Aβ aggregation 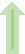 TBARS (Mishra and Palanivelu, 2008; Khuwaja et al., 2011; Tang and Taghibiglou, 2017)
*Vitis vinifera*	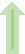 Formation of amyloid plaques, Tau tangles and oxidative stress Anti-inflammatory, anti-acetylcholinesterase and anti-amyloidogenic (Mishrikoti et al., 2022)
*Vaccinium corymbosum L*	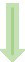 Death of dopaminergic cells (Ríos et al., 2016)
*Centella asiatica*	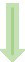 CAT and GSH 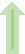 Oxidative stress, phospholipase A2 activity and MDA Inhibition of AChE, prevention of Aβ toxicity and improvement of age-related mood and cognitive disorders (Veerendra Kumar and Gupta, 2003; Rajamanickam and Manju, 2022)
*Commiphora whighitii*	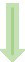 GSH 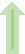 MDA and AChE Improve memory (Saxena et al., 2007)
*Achyranthes aspera*	Anti-inflammatory, antioxidant, anti-aging and free radical, AChE and BChE inhibition (Ayeni et al., 2022)
*Clitoria ternatea L.*	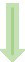 ACh and AChE inhibition (Hassan et al., 2014)
*Coriandrum sativum*	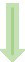 CAT, SOD and GSH Improving memory, antioxidant and anti-inflammatory (Akram and Nawaz, 2017; Bhattacharya et al., 2022)
*Withania somnifera*	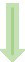 SOD- 1, CAT and GSH Stimulating axon and dendrite growth and preventing motor defects (Pérez-Hernández et al., 2016)
*Glycyrrhiza glabra*	Prevention of neuronal death due to Aβ and effective in treating HD and AD (Hassan et al., 2021)
*Salvia officinalis*	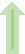 Tau hyperphosphorylation and caspase 3 activation Memory improvement, anti-inflammatory, antioxidant and AChE Inhibitor (Jivad and Rabiei, 2014)
*Hibiscus asper*	Improving memory and antioxidants (Ayeni et al., 2022)
*Mucuna pruriens*	Neuroprotective and contains natural L-dopa (Mishrikoti et al., 2022)
*Polygala tenuifolia*	Prevention of dementia and insomnia, inhibition of Aβ secretion and strengthening of the central cholinergic system (Jivad and Rabiei, 2014)
*Allium sativum*	Prevention of dementia, protection of dopamine levels and antioxidants (Bhattacharya et al., 2022)
*Tinospora cordifolia*	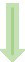 Learning, memory and acetylcholine synthesis (Hassan et al., 2014)
*Celastrus paniculatus*	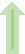 Noradrenaline and dopamine Improving learning and memory and having an antioxidant effect on the CNS (Jivad and Rabiei, 2014)
*Ginkgo biloba*	Inhibition of Aβ aggregation and neuroprotection 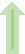 MDA (Malik et al., 2013)
*Galanthus nivalis*	Memory improvement, neuroprotection and AChE inhibition (Bhattacharya et al., 2022)

### 8.1 Flavonoids

Flavonoids are low molecular weight compounds obtained from various parts of plants such as roots, stems, and flowers, with over 6,000 types identified. They exhibit therapeutic properties for ND by reducing cell death caused by inflammation through the modulation of MAPK pathways, Akt, and NF-κB. Flavonoids inhibit the production of inflammatory cytokines, chemokines (Solanki et al., 2016; Martano et al., 2022), ROS and RNS, and inhibition of lipid peroxidation. These compounds have been shown to improve memory, increase neurogenesis, and suppress cytochrome c oxidase activity (Solanki et al., 2016; Prasanna and Upadhyay, 2021; Martano et al., 2022). Additionally, flavonoids have protective effects on PD and HD, preventing the spread of Aβ peptides and neurotoxic aggregations (Ramirez-Nuñez et al., 2018).

Specific flavonoids like baicalein, catechin, epigallocatechin-3-gallate (EGCG), fisetin, genistein, quercetin, and wogonin modulate neuroinflammation and reduce prostanoids levels. Genistein and silibinin inhibit AChE and BChE (Prasanna and Upadhyay, 2021).

Quercetin, in particular, has therapeutic properties for AD by removing free radicals and reducing inflammation. Quercetin is also a competitive inhibitor of AChE and BChE and inhibits them in a dose-dependent manner. Quercetin reduces the level of AChE in the hippocampus and increases it in the synaptic space by preventing the degradation of ACh (Testa et al., 2014; Khan et al., 2019; Liao et al., 2022).

Naringin is a member of the flavonoid group that is derived from various *citrus* fruits and *Artemisia selengensis* and it decreases the levels of IL-1β, TNF-*α*, malondialdehyde (MDA) and AChE and increases the levels of CAT, superoxide dismutase (SOD) and glutathione (GSH). This flavonoid improves mitochondrial and redox activity in the cerebral cortex and hippocampus (Sachdeva et al., 2014).

### 8.2 Phenols

Phenols are characterized by one or more aromatic rings with hydroxyl groups (Chandran and Abrahamse, 2020) and are part of a group of natural compounds known as polyphenols. These compounds possess anti-inflammatory, antioxidant, and anti-amyloid properties superior to synthetic compounds as part of a healthy diet (Velander et al., 2017). Phenolic compounds inhibit the secretion of IL-1β and TNF-*α*, induction of iNOS, production of NO, NADPH oxidase, and ROS while regulating the inhibition of pro-inflammatory transcription factors like NF-κB (Sharifi-Rad et al., 2022). Ellagic acid, a polyphenol derived from various plants (such as *Rosa rugosa, Rubus chamaemorus, Rubus ursinus × Rubus idaeus, Rubus allegheniensis,* and *Rubus fruticosus*) increases SOD levels, improves memory, inhibits tau hyperphosphorylation, and prevents Aβ toxicity (Ahmadi and Javid, 2023). It also reduces Aβ plaques in the cingulate cortex, hippocampus, and entorhinal cortex (Rezai-Zadeh et al., 2008). Curcumin, another polyphenol, improves memory, prevents the progression of AD, and exhibits anti-Aβ and anti-inflammatory properties, reducing the inflammatory response caused by Aβ in microglia. This polyphenol inhibits the oxidative stress caused by Aβ by increasing SOD and CAT, maintaining the level of GSH and reducing MDA. In addition, Curcumin inhibits AChE in the cortex and striatum with a mechanism similar to AD drugs (Hamaguchi et al., 2010; Tang and Taghibiglou, 2017; Chen et al., 2018).

### 8.3 Alkaloids

Alkaloids are compounds found in plants, mainly flowering plants, containing carbon, hydrogen, nitrogen, and, in most cases, oxygen. These compounds have anti-amyloid, anti-inflammatory, antioxidant, and neuroprotective properties, making them suitable and safe for treating neurodegenerative diseases (Hussain et al., 2018; Bakrim et al., 2022).

The alkaloid galantamine derived from *Amaryllidaceae* plants suppresses cytotoxicity and Aβ accumulation while stimulating ACh receptors. Juliflorine alkaloid derived from *Prosopis juliflora* leaves inhibits AChE and BChE (Hussain et al., 2018; Bakrim et al., 2022).

Berberine derived from the plants *Argemone Mexicana, Berberis aquifolium, Berberis vulgaris*, improves cognitive and motor skills and reduces levels of mutant protein huntingtin (Htt), NF-κB, α, IL 6, IL-8 and oxidative stress caused by ROS and RNS in HD patients (Singh et al., 2022).

Alkaloid huperzine-A decreases the levels of mutant protein Htt, ROS, MDA, TNF-α and AChE and increases SOD, CAT and Glutathione Peroxidase (Gpx) in HD patients (Subaraja et al., 2024).

Alkaloid derived from *Piper longum*, improves motor skills, increases dopaminergic neurons, SOD and GSH and decreases MDA level in patients with PD (Bi et al., 2015).

Alkaloids derived from the *Crossyne flava,* plant improve the morphology of neurons, inhibit ROS and apoptosis, and increase the level of adenosine triphosphate activity (ATP) in patients with PD (Omoruyi et al., 2021).

### 8.4 Terpenes

Terpenes represent the largest and most diverse group of secondary metabolites, consisting of simple hydrocarbons with multiple isoprene units. They exhibit various beneficial properties such as anti-cancer, anti-hyperglycemic, anti-inflammatory, antioxidant, immune-modulating, and anti-cholinesterase effects. Terpenes are known for their neuroprotective properties (Lai Shi Min et al., 2022; Bakrim et al., 2022). The terpene derived from *Alpinia oxyphylla* Miq has a neuroprotective effect and aids in synthesizing and releasing neurotransmitters from neurons. The terpene derived from *G. repens* has an inhibitory effect on AChE and BChE. Terpenes derived from *Nepeta obtusicrena* inhibit AChE and exhibit therapeutic properties for AD (Bakrim et al., 2022).

## 9 Problems of using medicinal plants

The poor uptake of certain herbal compounds has posed challenges in their treatment applications. For instance, the polyphenol curcumin exhibits limited absorption and bioavailability in AD treatment (Abhishek et al., 2023). The BBB is a semipermeable boundary consisting of endothelial cells, pericytes, astrocytes, and the basement membrane, serving as a protective interface between the CNS and peripheral circulation (Sahni et al., 2011; Nguyen et al., 2021; Bhattacharya et al., 2022). This barrier impedes the delivery of treatments to brain neurons (Shabbir et al., 2020).

Suitable approaches to enhance bioavailability and traverse the BBB should be explored. To this end, employing innovative techniques like combining plant compounds with nanoparticles proves viable (Abhishek et al., 2023).

## 10 Nanoparticles

Nanoparticles (NPs) are sizes ranging from 10 to 100 nm, categorized into ceramic, metal, semiconductor, carbon-based, lipid-based, and polymer groups based on their properties and structures. NPs possess small size, high reactivity, and a substantial surface-to-volume ratio (Saratale et al., 2018; Fakhri et al., 2022). They exhibit stability in the body, efficient cellular uptake, and the ability to neutralize superoxide anion and H_2_O_2_, and are applications in wound dressing (Xing et al., 2014; Ko et al., 2022). Among the most utilized NPs in AD diagnosis and treatment are gadolinium NPs, Selenium NPs (SeNPs), AuNPs, polymeric NPs, and protein- and polysaccharide-based NPs (Gupta et al., 2019).

**Figure 2:**
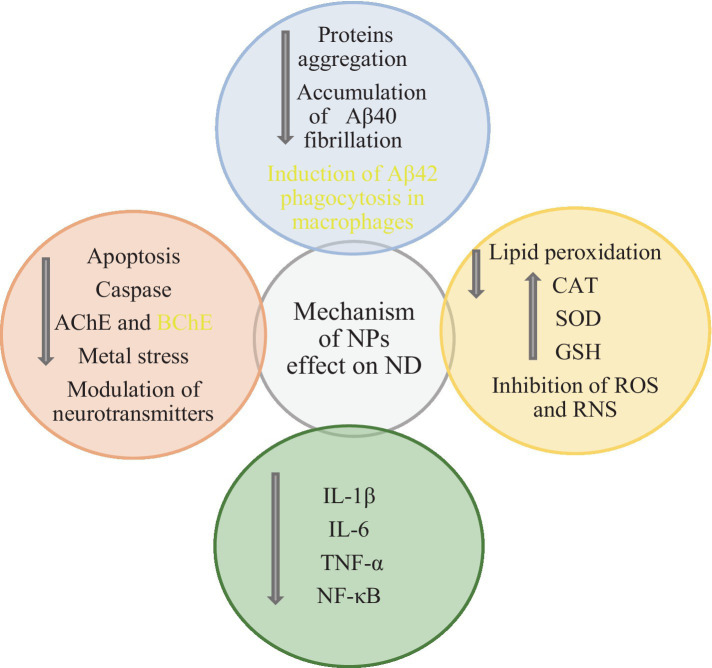
Anti-apoptotic, antioxidant, anti-inflammatory, anti-cholinesterase effects and other mechanisms of NPs in ND (Ovais et al., 2018; Fakhri et al., 2022; Woon et al., 2022; Behera et al., 2023).

The attributes of NPs, such as extensive surface area, high cellular uptake capability, and prolonged circulation in the bloodstream, facilitate their passage through the BBB, enabling effective drug delivery to the brain. NPs target specific molecular mechanisms based on the type of disease, addressing cells or intracellular and extracellular molecules like Aβ plaques (Zhang et al., 2013; Shabbir et al., 2020; Martano et al., 2022). NPs are crucial in managing oxidative stress, apoptosis, and neuroinflammation in ND (Fakhri et al., 2022; Woon et al., 2022) (Figure 2).

NPs impact amyloid fibrillation and the degradation of mature protein fibrils through six mechanisms: 1. Enhancing bioavailability, leading to the dispersion of insoluble molecules in water and enhancing the stability of unstable chemical molecules; 2. They are inhibiting protein aggregation during synthesis, 3. Facilitating high cellular uptake, 4. Leveraging the multivalence of NPs to enhance binding to protein aggregates, 5, mitigating toxicity induced by protein fibrils, 6 and ensuring precise targeting within the brain (Pradhan et al., 2018). Cationic NPs are internalized by cells through uptake-mediated endocytosis, highly hydrophilic NPs through receptor-mediated endocytosis, small hydrophilic NPs through the paracellular pathway, and small lipophilic NPs through passive diffusion via intercellular pathways (Shabbir et al., 2020). NPs can be absorbed through nerve terminals in the airway epithelium and transported to CNS axons. Additionally, they can reach the CNS via the olfactory bulb nerves (Sahni et al., 2011).

Various methods exist for synthesizing NPs. Physical methods encompass mechanical milling, laser ablation, sputtering, plasma arching, and chemical etching. In contrast, chemical methods include the sol–gel method, electrolytic deposition, chemical vapor deposition, microemulsion route pyrolysis, and green synthesis methods involving microorganisms, enzymes, and plant extracts (Garg et al., 2021).

## 11 Green synthesis

Synthesizing NPs via chemical methods can be toxic, while physical methods require high energy consumption. Both physical and chemical methods raise environmental concerns, making the green synthesis method appealing due to its safety, environmental friendliness, and low toxicity advantages (Rai et al., 2013; Saratale et al., 2018; Mikhailova, 2021). Biogenic nanoparticles can be readily functionalized with targeting ligands or therapeutic agents, facilitating precise targeting and delivery to cancer cells (Chaturvedi et al., 2023).

Key factors like temperature, time, reactant concentration, environmental conditions, pore size, and pH significantly influence the morphological properties of NPs. For instance, physical and chemical methods typically operate at high temperatures (> 350°C for physical methods and < 350°C for chemical methods), whereas the green synthesis method occurs at lower temperatures (≤ 100°C) (Bahramikia and Izadi, 2023). In green synthesis, photoautotrophic eukaryotes like microbes, algae, and plants can be utilized. Employing microorganisms for green synthesis is viable due to its safety and cost-effectiveness. Microorganisms exhibit selective metal ion absorption and can function under various ionic, temperature, and pH conditions. Different microorganisms, including fungi, bacteria, and yeasts, can be us for green synthesis (Saratale et al., 2018).

Algae, serving as blue photoautotrophs, are primary producers in green synthesis environments. They have been instrumental in synthesizing AuNPs, AgNPs, CuNPs, ZnNPs, ZnONPs, and CuONPs. For instance, *Sargassum wightii* algae has been utilized for extracellular AuNP synthesis (Saratale et al., 2018).

Using plant extracts for green synthesis of metal and metal oxide NPs is one of the simplest methods for green synthesis. Plant extracts containing ketones, aldehydes, flavonoids, amides, terpenoids, phenols, carboxylic acids and ascorbic acids are used for green synthesis (Bhattacharya et al., 2022). NPs synthesized through green methods find applications in treating various diseases like cancer, diabetes, and bacterial infections, showcasing antioxidant and anti-inflammatory properties (Bahramikia and Izadi, 2023) Ag NPs and Au NPs showed promising results against a human colon cancer cell line. These NPs reduced the proliferation of a cancer cell line by generating a large amount of intracellular ROS (Chaturvedi et al., 2020). Oyster mushroom mediated bimetallic Au-Pt nanoparticles exhibited apoptotic activity on the human colon cancer cell line in a dose-dependent manner (Chaturvedi et al., 2021a). In addition, oyster mushroom mediated Au–Pt–Ag trimetallic nanoparticles successfully killed triple-negative breast cancer cells with superior IC50 values (Chaturvedi et al., 2020).

### 11.1 Metal NPs and metal oxide synthesized with medicinal plants and their compounds

Numerous metal NPs have been developed for drug delivery, with AuNPs and AgNPs being the most prevalent in biomedicine (Jabbar et al., 2018). Metal NPs and metal oxides exhibit anti-inflammatory properties (Xing et al., 2014; Ko et al., 2022). Notably, AuNPs, AgNPs, CuNPs, SeNPs, ZnONPs, magnesium oxide NPs (MgONPs), Cerium oxide NPs (CeONPs), and FeONPs possess anti-inflammatory attributes and demonstrate effectiveness in AD treatment (Fakhri et al., 2022). AuNPs inhibit tau hyperphosphorylation, alter the secondary structure of Aβ, enhance memory, reduce H_2_O_2_, and elevate CAT, SOD, and GSH levels (Lee et al., 2014; dos Santos Tramontin et al., 2020). Silver, gold, and many other nanoparticles effectively prevent progressive neurodegeneration in PD (Chaturvedi et al., 2021a). Various studies have demonstrated the potential benefits of NPs synthesized with natural extracts in treating neurodegenerative diseases (Table 2).

**Table 2:** Metal NPs treatments synthesized by green method for ND.

Medicinal plants and metal NPs	Mechanism of effect
*Terminalia arjuna* bark + AuNPs	Inhibit the DPPH free radical and AChE, and BChE, preventing protein misfolding and fibrillation, formation of Aβ plaque (Suganthy et al., 2018)
*Aquilegia pubiflora +* AgNPs.	Inhibitory effects on AChE, BChE, COX-1, and COX-2 enzymes 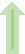 Total reduction power (TRP), total antioxidant capacity (TAC), ABTS, DPPH, and free radicals scavenging assays (FRSA) (Jan et al., 2021b)
*Prosopis cineraria* (L.) + ZnONPs	Inhibitory effects on DPPH and AChE and improved memory 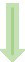 SOD, CAT, and GPx (Yadav et al., 2018)
*Rosa* petal + AgNPs	Inhibit Aβ aggregation, protecting astrocytes from toxicity (Rauf et al., 2022)
*N. khasiana* leaf + AgNPs	Inhibit ROS production, and improve oxidative stress 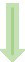 Memory, activate mitochondria (Zhang et al., 2020)
*Lampranthus coccineus* and *Malephora lutea +* AgNPs	Antioxidant AChE and MDA 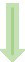 GSH (Youssif et al., 2019)
*Convolvulus Pluricaulis* + FeONPs	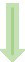 Learning, memory and CAT 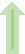 AChE and MDA (Poka et al., 2017)
*Millettia pinnata* flowers + AgNPs	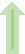 AChE and BChE (Rajakumar et al., 2017)
*Paeonia moutan* root + AuNPs	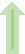 IL-1β, IL-6, TNF-*α*, ROS, NO, COX-2, iNOS, and prostaglandin E2 (PGE2) 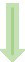 Dopamine levels, tyrosine hydroxylase enzyme activity, motor coordination, and step length distance (Xue et al., 2019)
Curcumin + Fe_3_O_4_ carbon dotsNPs	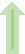 Inhibit ROS production and formation and aggregation of Aβ42 fibrils Aβ-induced toxicity (Kuang et al., 2020)
*Sabal blackburniana* fruit and leaf + ZnONPs	Inhibition of AChE (El-Hawwary et al., 2021)
*Bacopa monnieri +* platinum NPs (PtNPs)	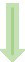 GPx, SOD, CAT, and GSH, dopamine, dihydroxyphenylacetic acid and homovanillic acid 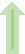 MDA and ROS (Nellore et al., 2013)
*Aquilegia pubiflora* leaf + ZnO-NPs	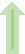 Inhibited AChE and BChE TRP, TAC, FRSA, and DPPH secretory phospholipase A2 (sPLA2), 15-LOX, COX-1, and COX-2 (Jan et al. 2021a)

### 11.2 Polymeric NPs synthesized with medicinal plants and their compounds

Polymeric drug carriers with nanometer sizes are being explored for their advantageous properties, including high drug-carrying capacity, stability, solubility, targeted tissue absorption, controlled drug release, and suitability for hydrophilic and hydrophobic compounds (Naseri et al., 2022). These polymeric NPs can traverse the BBB and hold promise for treating neurodegenerative diseases such as AD (Gupta et al., 2019). Various studies have investigated the application of polymeric NPs for treating neurological diseases, including AD (Table 3).

**Table 3:** Polymeric NPs treatments synthesized by green method for ND.

Medicinal plants and polymeric NPs	Mechanism of effect
EGCG from green tea + NPs derived from dopamine functionalized polysuccinimide	Clearance of soluble mutant huntingtin Inhibited Aβ fibrils and blocked polyglutamine of cells 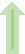 Insoluble mutant Htt and Aβ-induced toxicity (Debnath et al., 2016)
Curcumin + chitosan-bovine serum albumin NPs	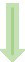 Promoted Aβ42 phagocytosis 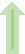 Macrophage polarization IL-6, TNF-α, and TLR4, and phosphorylation of ERK, JNK, p38, and NF-κB (Yang et al., 2018)
Curcumin + ApoE3-mediated poly (butyl) cyanoacrylate NPs	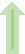 Protective Aβ-induced toxicity and antioxidant and anti-apoptotic ROS and caspase 3 induced by Aβ (Mulik et al., 2010)
Curcumin + polylactic coglycolic acid NPs (PLGA NPs)	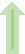 Size of amyloid aggregates Antioxidant activity, neutralizing DPPH free radicals up to 60% bind to amyloid aggregates and reduce their size (Mathew et al., 2012)
Curcumin + PLGA NPs	Facilitated sequestration and removal of Aβ, prevented the reduction of the number of synapses, and inhibited the activation of NF- κB (Barbara et al., 2017)

### 11.3 Other NPs synthesized with medicinal plants and their compounds

Other NPs synthesized using medicinal plants and their compounds have shown promising effects in various studies (Table 4).

**Table 4:** Other NPs NPs treatments synthesized by green method for ND.

Medicinal plants and other NPs	Mechanism of effect
*Areca Catechu* L leaf + hydroxyapatite NPs	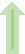 AChE and BChE (Pradeep et al., 2022)
Curcumin-encapsulated solid lipid NPs	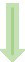 GSH, SOD, NADH dehydrogenase, cytochrome c oxidase, mitochondrial F1F0 synthase activity, average speed, and motor activity 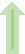 ROS, MDA, and walking angle (Sandhir et al., 2014)

## 12 Conclusion

**Figure 3:**
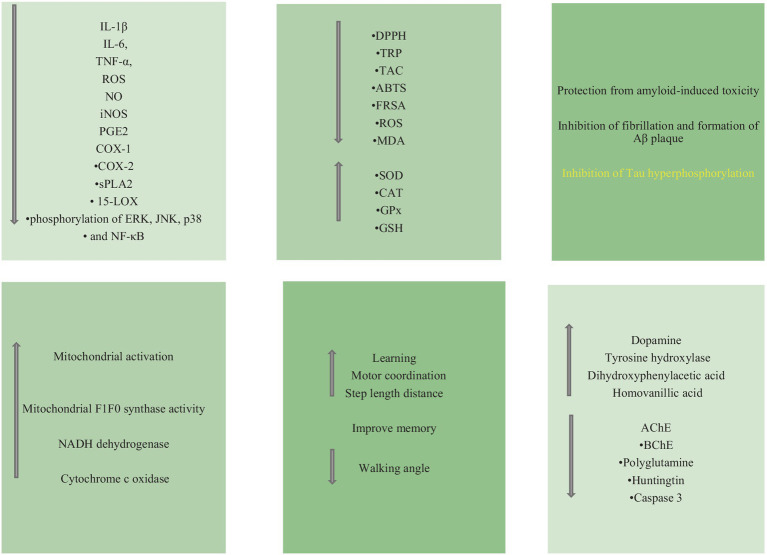
The most important events occurring in ND after treatment with NPs synthesized by green method.

Bioactive molecules such as sterols, polyphenols, lignans, flavonoids, alkaloids, triterpenes, and tannins, which are abundant secondary metabolites in the diet, have been utilized to treat CNS-related diseases. These secondary metabolites have demonstrated inhibitory effects on Aβ, toxicity induced by AChE, BChE, oxidative stress, and neuroinflammation (Gul et al., 2021; Bakrim et al., 2022). The unique characteristics of NPs, including their large surface area, high cellular uptake capacity, and prolonged presence in the bloodstream, enable them to traverse the BBB and efficiently deliver drugs to the brain. NPs can target specific molecular mechanisms based on the type of disease, addressing cellular, intracellular, or extracellular targets such as Aβ plaques (Zhang et al., 2013; Shabbir et al., 2020; Martano et al., 2022). Using plant extracts for green synthesis of metal and metal oxide NPs is one of the simplest methods for green synthesis. Plant extracts containing ketones, aldehydes, flavonoids, amides, terpenoids, phenols, carboxylic acids and ascorbic acids are used for green synthesis (Bhattacharya et al., 2022). These green-synthesized NPs have been employed in treating various diseases, including cancer, diabetes, and bacterial infections, and have demonstrated antioxidant and anti-inflammatory properties (Bahramikia and Izadi, 2023). Additionally, metal NPs and metal oxides exhibit anti-inflammatory properties (Xing et al., 2014; Ko et al., 2022). Polymeric NPs, known for their ability to cross the BBB, hold the potential for treating neurodegenerative diseases such as AD (Gupta et al., 2019). Furthermore, NPs synthesized via the green synthesis method offer diverse mechanisms for treating neurodegenerative diseases (Figure 3).
